# A Footnote to the Evolution of Digits

**DOI:** 10.1371/journal.pbio.1001774

**Published:** 2014-01-21

**Authors:** Mary Hoff

**Affiliations:** Freelance Science Writer, Stillwater, Minnesota, United States of America

Half a billion years ago, the first four-legged land animal crawled out of the sea onto dry land. How did the limbs that creature crawled on evolve from the fins of its fishy ancestors? This question has long intrigued biologists.

**Figure 1:**
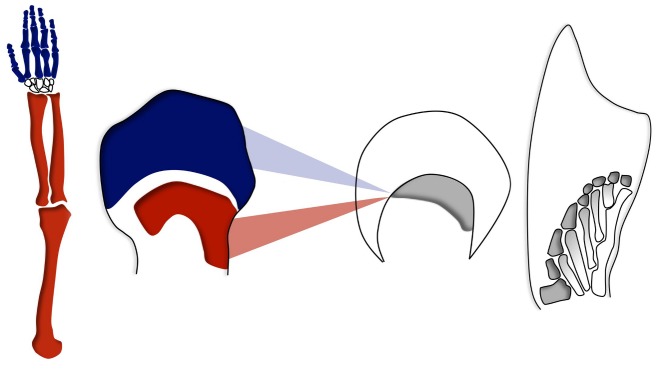
Hox gene expression in bones from tetrapod limbs. Bones in a tetrapod limb, shown here in a human arm (left), are produced following two waves of Hox gene activation during the early development of the limb (red and blue, middle left). At a comparable developmental stage, fish fins show only one domain of Hox gene expression (middle right), which subsequently generates complex bony patterns. Is this fish domain homologous to the proximal or to the distal expression domain in tetrapods? Can the underlying regulatory mechanisms help establish such evolutionary relationships? A recent study in *PLOS Biology* addresses these questions.

Fossil records suggest that tetrapod legs evolved step by step from fins, and comparative gene expression studies have provided some insights into how mutation and natural selection derived long limb bones from fin precursors. But the evolutionary path that lies between the structural elements (radials) of fish fins and the toes and fingers of tetrapod digits has remained obscured. Are tetrapod digits homologous to fish radials? Did the genetic capacity for digit differentiation exist in fish ancestors, or is it unique to tetrapods? In their recent *PLOS Biology* article, Denis Duboule, Joost M. Woltering, and colleagues shed new light on these questions from a comparative analysis of the regulatory mechanisms that control when and where certain members of the Hox gene family are turned on and off in zebrafish fins and mouse limbs.

Two Hox gene clusters, *HoxA* and *HoxD*, are known to function in patterning the developing vertebrate limb. In land animals, but not in fish, *HoxD* has what is known as a “bimodal expression pattern,” meaning that one subset of *Hoxd* genes directs the development of the long bones on the proximal (body) side of the wrist or ankle, while another subset directs the development of the long bones on the distal side (i.e., the digits). This bimodal expression pattern is due to the preferential interaction of the 3′ and 5′ genes in these Hox clusters with flanking regions of DNA on their own side of the cluster. These flanking regions contain non-genic DNA in which are located so-called enhancers—DNA loci that control the transcription of genes. On the 3′ side of the HoxA and Hox D clusters sit the proximal enhancers, which regulate the expression of proximal genes in the cluster in the proximal part of the limb, and on the 5′ side are the distal enhancers, which regulate the expression of distal genes in the cluster, leading to the segregated pattern of 3′ and 5′ genes. Duboule and colleagues decided to find out whether the *HoxA* cluster shares this regulatory characteristic with *HoxD*, reasoning that if it does, that the bimodality arose before the Hox clusters duplicated, indicating also that the regulatory capacity to form digits was in place before land animals' evolutionary emergence from the sea. Such a finding would lend strength to the argument for homology between digits and fin radials.

By looking at the expression patterns of *Hoxa* genes in mouse embryos and by comparing them with the expression patterns of *Hoxd* genes, the researchers determined that the *HoxA* gene cluster does indeed exhibit biomodality, with one regulatory module directing the development of the digits and another orchestrating the development of the proximal segment of the limb. Although they noted some differences in the details of the bimodal expression of *HoxA* and *HoxD* genes, the researchers concluded that this pattern was common to both these Hox clusters and therefore predated the evolution of tetrapods from their fish ancestors.

If both *HoxA* and *HoxD* clusters share the bimodality that is associated with the differential regulation of the proximal and distal development of tetrapod limbs, is the same true of the Hox clusters that direct fin development in fish? By looking at the interaction profiles of Hox genes in zebrafish embryos, the researchers discovered a partitioning pattern similar to that seen in the mouse, in which Hox genes located at the edge of the clusters tended to interact more with their nearest flanking DNA regions while those in the middle of the cluster interacted with the flanking regions on both sides, confirming the existence of this bimodal pattern. Their findings support the idea that the chromatin structure that underlies this regulatory mechanism existed before the evolution of tetrapod limbs and that digits, therefore, may be homologous to distal fin structures in fish.

The team then inserted (in separate experiments) zebrafish *HoxA* and *HoxD* clusters, together with their flanking 5′ regions, into mice. Surprisingly, in the resulting transgenic mouse embryos, zebrafish Hox gene expression was specific to the proximal and not distal (digit-associated) developing limb tissues. On the basis of these findings, the authors conclude that the bimodal regulatory landscape that controls *HoxA* and *HoxD* expression was indeed in place before fish and tetrapods diverged, and that the subsequent evolution of novel enhancers allowed it to be repurposed to bring about the development of tetrapod digits.

Returning to the original question: does this make fin radials and digits homologous? That, the authors decided, depends on definitions. Their findings clearly demonstrate that fish have both the genes and the regulatory architecture needed to form digits. However, they also show that the development of digits depends on additional genetic alterations occurring in the context of that preexisting regulatory landscape. Duboule and colleagues suggest that although fish radials are not homologous to digits in the classical sense, biologists should consider thinking in terms of regulatory circuitries rather than expression patterns when considering whether traits have arisen from a common ancestral characteristic.

**Woltering JM, Noordermeer D, Leleu M, Duboule D (2014) Conservation and Divergence of Regulatory Strategies at* Hox* Loci and the Origin of Tetrapod Digits** doi:10.1371/journal.pbio.1001773

